# The Effect of Calcium Chloride on Growth, Photosynthesis, and Antioxidant Responses of *Zoysia japonica* under Drought Conditions

**DOI:** 10.1371/journal.pone.0068214

**Published:** 2013-07-02

**Authors:** Chengbin Xu, Xuemei Li, Lihong Zhang

**Affiliations:** 1 School of Environmental Science, Liaoning University, Shenyang, China; 2 College of Chemistry and Life Science, Shenyang Normal University, Shenyang,China; Manchester University, United Kingdom

## Abstract

Few attempts have been made to study the alleviating effects of signal molecules on zoysiagrass ( *Zoysia* *japonica* ) under drought stress. Calcium chloride has been shown to ameliorate the adverse effects of drought stress on many plants. It is necessary to investigate how to enhance drought tolerance of zoysiagrass using calcium chloride. The study elucidated the effects of calcium chloride on zoysiagrass under drought conditions by investigating the following parameters: biomass, chlorophyll (Chl) content, net photosynthetic rate (Pn), chlorophyll fluorescence, antioxidant enzymes, proline content, and malondialdehyde (MDA) content. Experimental conditions consisted of an aqueous CaCl_2_ solution at 5, 10, and 20 mM sprayed on zoysiagrass leaves for 3 d, following by an inducement of drought conditions by withholding water for 16 d. Under drought conditions, all CaCl_2_ pretreatments were found to increase the above-ground fresh biomass, as well as below-ground fresh and dry biomass. The resulting Chl (a, b, a+b) contents of the 5 and 10 mM CaCl_2_ pretreatment groups were higher than those of the control. In the later stages of drought conditions, the chlorophyll fluorescence parameter Fv*/*Fm was higher in leaves treated with 10 mM CaCl_2_ than in the leaves of the other two treatment groups. Zoysiagrass pretreated with 10 mM CaCl_2_ possessed both the maximum observed Pn and antioxidant enzyme activities. Meanwhile, lower MDA and proline contents were recorded in the plants pretreated with 5 and 10 mM CaCl_2_ under drought conditions. As a whole, the drought tolerance of zoysiagrass was improved to some extent by the application of a moderate calcium concentration.

## Introduction

Drought is a major environmental factor that adversely affects the productivity and survival of plants [1]. Plants that experience drought conditions often appear serious physiological and biochemical dysfunctions, including: reduction in turgor, growth, photosynthetic rate, and stomatal conductance, as well as damage to various cellular components [2,3]. Plants utilize various mechanisms to adapt to and withstand drought conditions [4]. For example, in plants subjected to drought, the generation of a variety of active oxygen species (ROS) has been observed. These include: superoxide anion (O^-^_2_), hydrogen peroxide (H_2_O_2_), and hydroxyl radical (·OH), all of which can cause oxidative damage to plants, including membrane lipid peroxidation [5]. When plant capacity to detoxify ROS increases, an increase in drought tolerance can be observed [6,7]. The rapid accumulation of proline, an osmoregulation substance in cells, constitutes another biochemical mechanism in plants that acts to achieve drought tolerance [8,9].

Various physiological practices have been applied to alleviate the adverse effects of a water deficit on normal plant functioning. For example, researchers have applied the following in an effort to improve plant growth under drought conditions: calcium ion [10,11], plant growth regulators [12–15], and other substances [16–18].

Importantly, calcium is involved in the regulatory mechanisms that plants activate to adjust to adverse environmental conditions of drought [10,11,19], heat [20,21], cold [22,23], salt [24,25], and heavy metal [26,27]. Further, calcium has been shown to ameliorate the adverse effects of water stress on plants [28], and is involved in signaling anti-drought responses [10]. Calcium appears to play a central role in many defense mechanisms that are induced by drought, and Ca^2+^ signaling is required for the acquisition of drought tolerance or resistance [29].

*Zoysia* *japonica* (zoysiagrass) is an important turfgrass species in East Asia, and particularly in China, Korea, and Japan [30]. In turfgrass management, drought constitutes a major factor limiting grass growth. Some researchers have studied the morphological and physiological responses of zoysiagrass to drought conditions [3,31–33]. However, to our knowledge, few attempts have been made to study the alleviation of such negative effects to drought stress. It is therefore necessary to investigate how to enhance drought tolerance using signal molecules.

As a result, we studied the effects of calcium applications on the physiological and biochemical mechanisms in leaves of *Z* *. japonica* under drought conditions. In the present study, we analyzed plant growth, as well as photosynthesis, chlorophyll fluorescence, lipid peroxidation, proline content, and activities of antioxidant enzymes.

## Materials and Methods

### Plant materials and treatments

Zoysiagrass plants were collected from 1-year-old turfgrass plots at the Ecological Research Center, Liaoning University. Plants were grown in plastic pots (diameter: 13 cm diameter, depth: 14.5 cm) filled with a mixture of topsoil and coarse river sand (1:1) in a greenhouse for one week (25°C day / 20 °C night, 16 hr/8 hr light / dark period, 800 µmol m^-2^ s^-1^ photosynthetically active radiation, and 75% relative humidity). The pots were then transferred to the experiment field and grown under natural conditions at the Liaoning University for one month. During this period, plants were watered to field capacity (≥ 75%) every 2 d and were fertilized once a week using compound fertilizer. Once the assay began, fertilization was stopped.

Plants were randomized and divided into four groups of 60 individuals. Aqueous calcium chloride solutions at 5, 10, and 20 mM containing 0.1% Tween 20 were sprayed on the leaves of three of the four groups until runoff thrice a day for 3 d. Control plants were sprayed with distilled water containing 0.1% Tween 20. Drought was induced through withholding water for one week. The soil water content then reached 7-8%, and maintained this degree until 16 d after CaCl_2_ application. Thus, the treatments were as follows: (1) Control: non-CaCl_2_ and drought, (2) 5 mM: 5 mM CaCl_2_ pretreatment and drought, (3) 10 mM: 10 mM CaCl_2_ pretreatment and drought, and (4) 20 mM: 20 mM CaCl_2_ pretreatment and drought. Each treatment was carried out in triplicate.

### Soil water content

After the treatment period was complete, the soil was taken from each pot and weighed immediately to obtain its wet weight. The soil was then dried for 48 hr at 90 °C, and wet and dry weights were then used to estimate soil water content (SWC). SWC was calculated as [(soil wet weight) - (soil dry weight)] / (soil wet weight) × 100.

### Estimation of plant biomass

The above-ground plants in the 13 cm diameter pots were clipped at 16 d after CaCl_2_ application. The roots in the pots were washed with distilled water. Above- and below-ground fresh and dry biomass weights were recorded, and dry biomass was obtained by oven-drying samples at 80 °C for 24 hr.

### Determination of Chl content and net photosynthetic rate

The Chl content was quantified using the method of Agrawal and Rathore [34]. Chl content was extracted from 0.1 g of leaf discs with 10 ml 80% acetone, and the absorbance of the solution was measured at 663 and 645 nm.

Between 10:00 and 12:00 on each sampling day, gas exchange by leaves (one leaf per plant, three plants per replicate) was measured with a portable photosynthesis system (Li-6400, Li-Cor, Lincoln, NE, USA). Net photosynthetic rate (Pn) was measured under ambient CO_2_ (370 µmol mol^-1^). Photosynthetic photon flux density (PPFD) was set at 800 µmol m^−2^ s^−1^ in the cuvette containing the leaf for Pn measurement.

### Measurement of chlorophyll fluorescence

Fluorescence parameters of intact leaves (three leaves per plant, three plants per replicate) were measured using Li-6400-40LCF (Li-Cor, Lincoln, NE, USA). The minimal chlorophyll fluorescence (*F*o) level when photosystem II centres are open was measured after applying a far-red pulse for 6 s. The maximal fluorescence (Fm) after 30 min of dark adaptation was measured after applying a saturating flash for 0.8 s. Maximal photochemical efficiency of PSII (Fv/Fm) was expressed as: Fv/Fm=(Fm-Fo)/Fm.

### Extraction and assay of antioxidant enzymes

Fresh leaf sample (0.5 g) was homogenized in 5 ml extraction buffer (0.1 M phosphate buffer pH 6.8) using a mortar and pestle on ice. The homogenate was then centrifuged at 12,000 × g for 15 min at 4 °C, and the supernatant was used as the crude extract for superoxide dismutase (SOD), catalase (CAT), and peroxidase (POD).

SOD activity was measured according to the method of Beyer and Fridovich [35]. SOD activity was assayed by measuring the ability of the enzyme in the crude extract to inhibit the photochemical reduction of nitroblue tetrazolium (NBT) by photochemically-generated superoxide radicals. One unit of SOD was defined as the amount of enzyme required to inhibit the rate of reduction NBT by 50% at 25 °C.

POD and CAT activities were assayed following the method of Chance and Maehly [36] with some modiﬁcation. The POD reaction solution contained 50 mM phosphate buffer (pH 7.8), 25 mM guaiacol, 200 mM H_2_O_2_, and the enzyme extract. Changes in absorbance of the reaction solution at 470 nm were determined. CAT activity was assayed in a reaction mixture containing 50 mM potassium phosphate buffer (pH 7.0, containing 0.1 mM EDTA), 200 mM H_2_O_2_, and the enzyme extract. The reaction was started with the addition of the enzyme extract, and the decomposition rate of H_2_O_2_ was followed at 240 nm.

### Estimation of MDA content

MDA content was determined using the method of Fu and Huang [37]. Fresh leaf sample (1.0 g) was homogenized with 4 ml of 0.1% (w/v) trichloroacetic acid (TCA) in an ice bath. The homogenate was centrifuged at 12,000 × g for 20 min, and the supernatant was used for lipid peroxidation analysis. A total of 4 ml of 0.5% thiobarbituric acid (TBA) in 20% TCA was added to 1 ml aliquot of the supernatant. The mixture was incubated in boiling water for 30 min. MDA content was then detected spectrophotometrically at 532 nm and corrected for nonspecific turbidity at 600 nm.

### Estimation of proline content

Free proline content was estimated using the method described by Bates et al. [38]. Fresh leaves (0.5 g) were extracted in 5 ml of 3% sulphosalicylic acid, and the homogenates were centrifuged at 3,000 × g for 20 min. A total of 2 ml of the supernatant was reacted with 4 ml of 2.5% acid ninhydrin reagent and 2 ml of glacial acetic acid in a test tube. After boiling the mixture in a water bath at 100 °C for 60 min, the reaction was stopped by cooling the tubes in ice bath for 5 min. The chromophore formed was extracted with 4 ml of toluene and mixed vigorously by vortexing for 0.5 min. Absorbance of the resulting organic layer was measured at 520 nm. The concentration of proline was estimated by referring to a standard curve for L-proline.

### Statistical analysis

All experiments were conducted with three replicates, and results were expressed as mean ± standard deviation (SD). All data were subjected to one-way analysis of variance (ANOVA) and LSD multiple comparison test (p < 0.05) using the SPSS statistical package.

## Results

### SWC during drought stress

**Figure 1:**
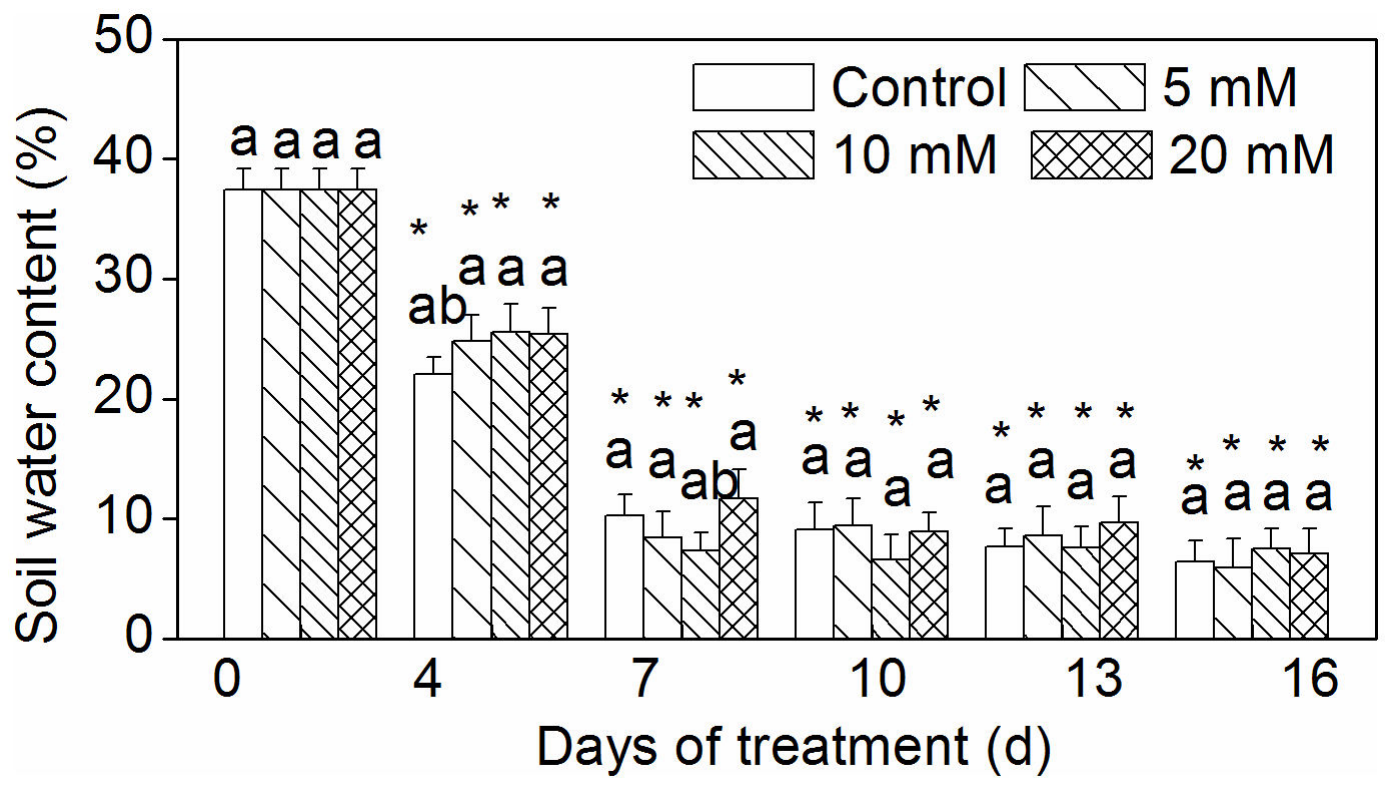
Soil water content during drought. Vertical bars represent standard deviations (n=3). The different letters indicate significant difference among different treatments on a given day (p < 0.05, LSD multiple test). The asterisks indicate significant difference between given day and 0 d at the same treatment (p < 0.05, LSD multiple test).

The SWC values observed during the treatment period are shown in Figure 1. During treatment, the SWC values for all CaCl_2_ pretreatments and the control significantly decreased to approximately 8% at 7 days after drought. The SWC values for all CaCl_2_ pretreatments and the control were not significantly different from one another, suggesting that the level of drought was the same for all treatments.

### Changes in above- and below-ground biomass

**Figure 2:**
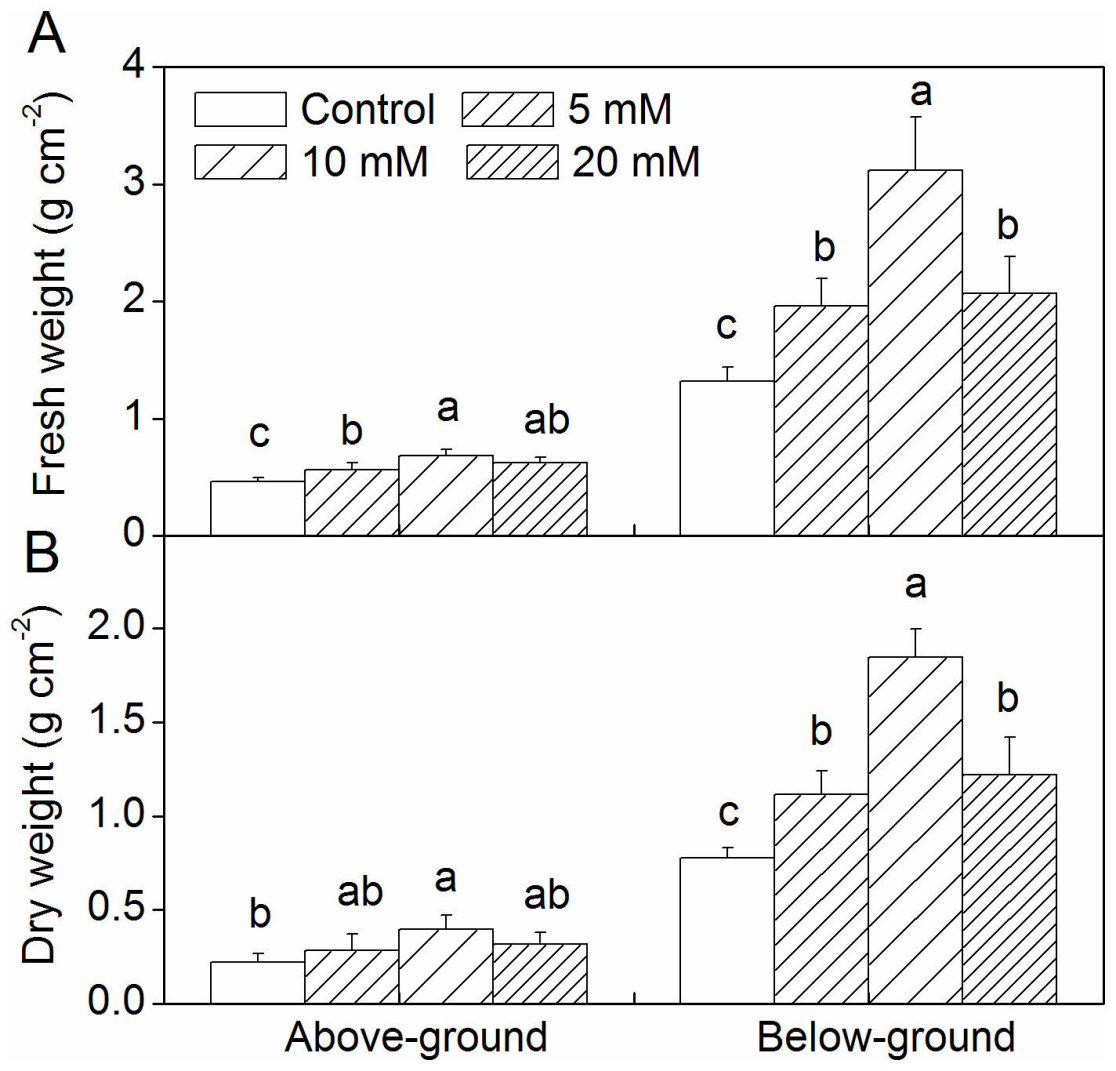
Effect of CaCl_2_ on above- and below-ground biomass of *Zoysia* *japonica* under drought. (A) Fresh weight; (B) Dry weight. Vertical bars represent standard deviations (n=3). The different letters indicate significant difference at p < 0.05 (LSD multiple test).

An increase in the above- and below-ground fresh biomass was observed for all CaCl_2_ pretreatment groups, and the greatest increase was observed for the 10 mM CaCl_2_ pretreatment group (Figure 2A). All CaCl_2_ pretreatment groups had below-ground dry biomass values that were higher than those of the control, while above-ground dry biomass increased in the 10 mM CaCl_2_ pretreatment group (Figure 2B).

### Changes in Chl content

**Figure 3:**
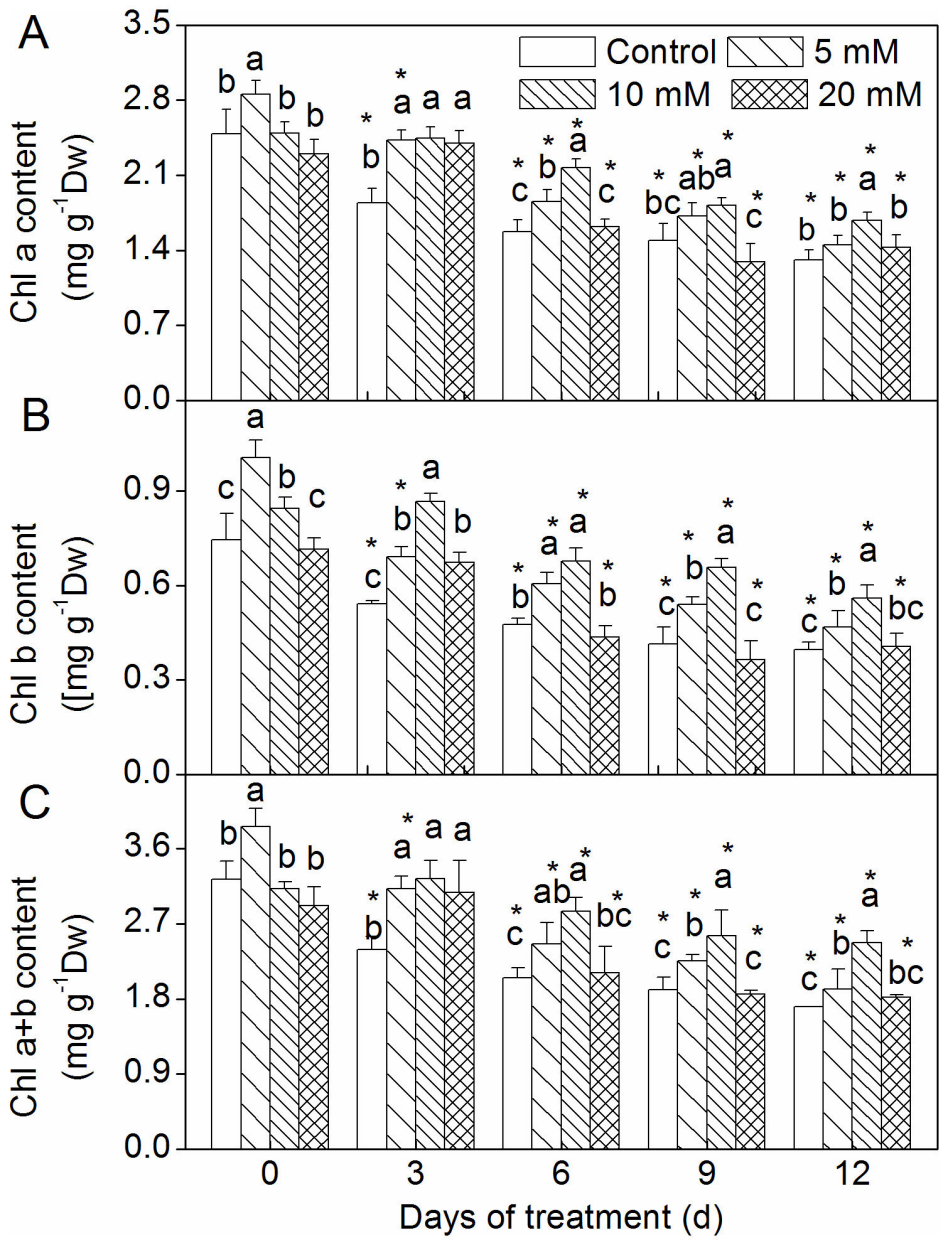
Effect of CaCl_2_ on chlorophyll content of *Zoysia* *japonica* leaves under drought. (A) Chl a content; (B) Chl b content; (C) Chl a+b content. Vertical bars represent standard deviations (n=3). The different letters indicate significant difference among different treatments on a given day (p < 0.05, LSD multiple test). The asterisks indicate significant difference between given day and 0 d at the same treatment (p < 0.05, LSD multiple test).

The Chl content (Chl a, Chl b and Chl a+b) values decreased with drought treatment (Figure 3). An increased Chl a content was observed for the 10 mM CaCl_2_ pretreatment group alone (Figure 3A). Meanwhile, Chl b and Chl a+b contents of the 5 and 10 mM CaCl_2_ pretreatment groups were higher than those of the control (Figure 3B, C), while Chl b and Chl a+b contents for the 10 mM CaCl_2_ pretreatment group was higher than those of the 5 mM CaCl_2_ pretreatment group.

### Changes in Pn and chlorophyll fluorescence

**Figure 4:**
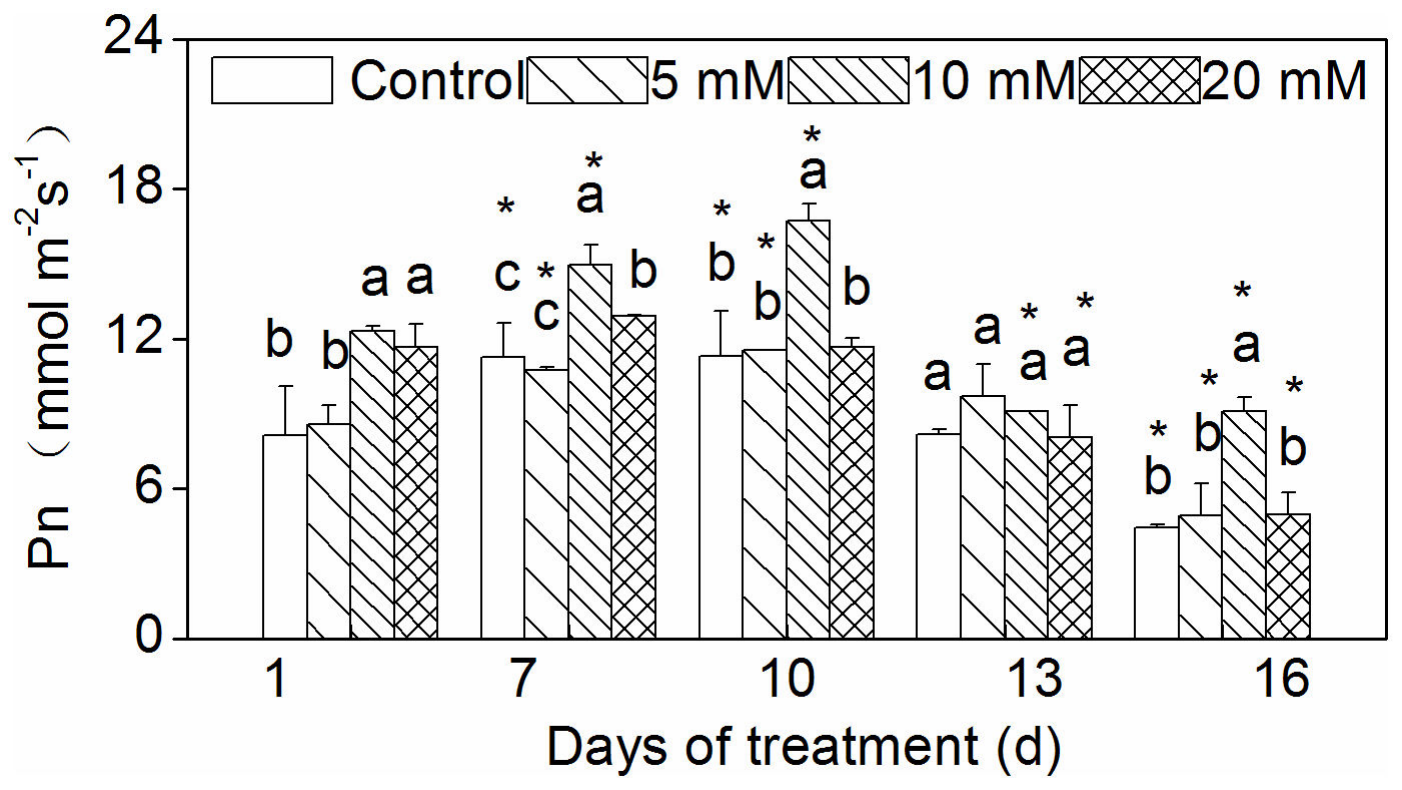
Effect of CaCl_2_ on net photosynthetic rate of *Zoysia* *japonica* leaves under drought. Vertical bars represent standard deviations (n=3). The different letters indicate significant difference among different treatments on a given day (p < 0.05, LSD multiple test). The asterisks indicate significant difference between given day and 0 d at the same treatment (p < 0.05, LSD multiple test).

Compared to the control, Pn of the 10 and 20 mM CaCl_2_ pretreatment groups significantly increased at the early stages of drought (from 1 to 7 days after drought) (Figure 4). At the later stages of drought, Pn of the 10 mM CaCl_2_ pretreatment group alone was positive, because it also exhibited an increased Pn compared to the control.

**Figure 5:**
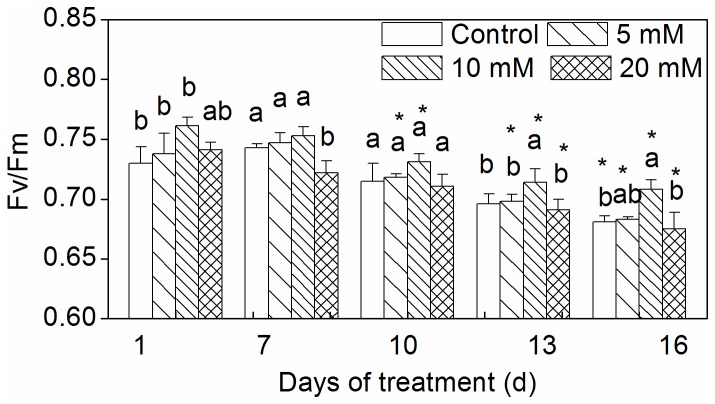
Effect of CaCl_2_ on Fv*/*Fm of *Zoysia* *japonica* leaves under drought. Vertical bars represent standard deviations (n=3). The different letters indicate significant difference among different treatments on a given day (p < 0.05, LSD multiple test). The asterisks indicate significant difference between given day and 0 d at the same treatment (p < 0.05, LSD multiple test).

The chlorophyll fluorescence parameter Fv/Fm ratio decreased as drought was prolonged. The Fv/Fm ratio was higher in leaves treated with 10 mM CaCl_2_ than in those of the other treatments (Figure 5).

### Changes in antioxidant enzymes activities

**Figure 6:**
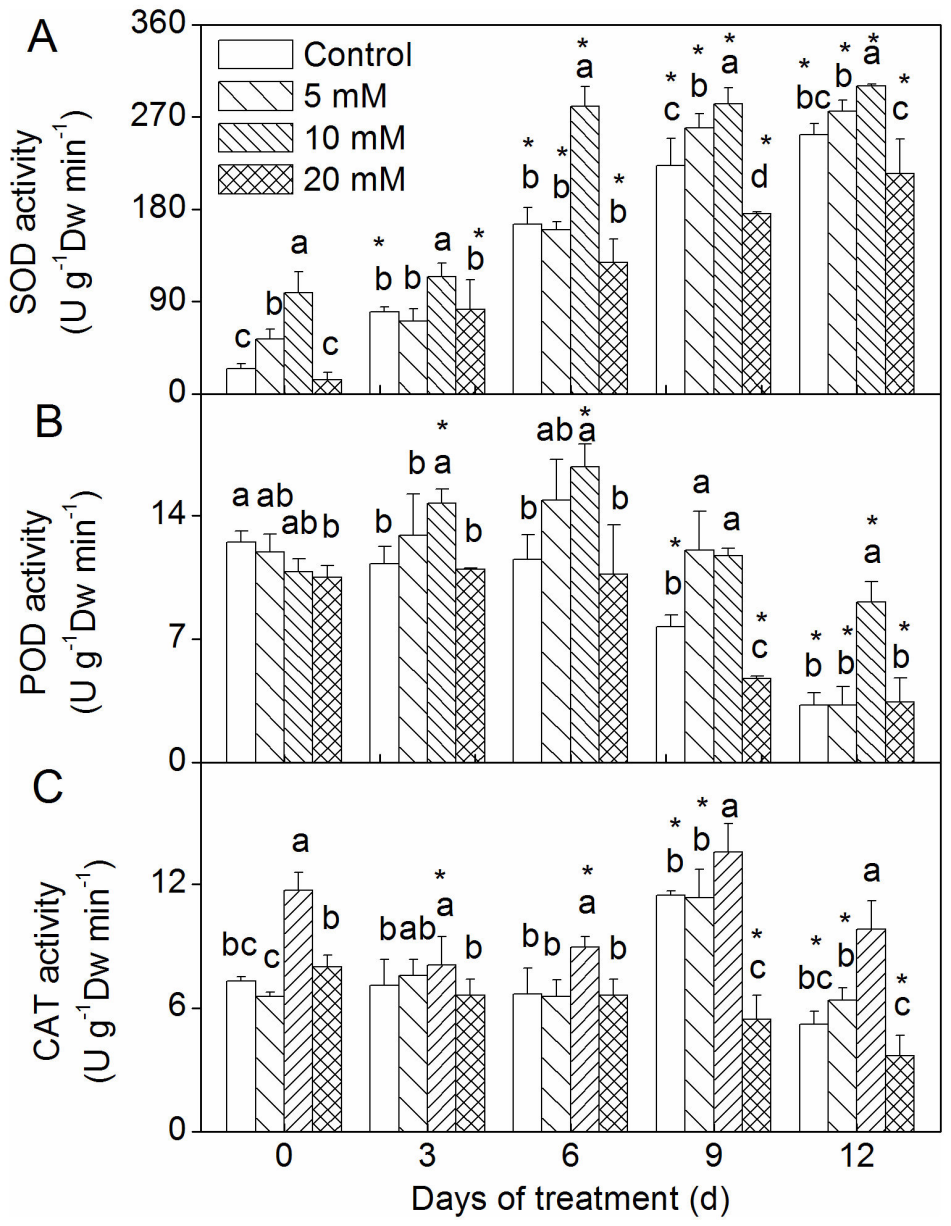
Effect of CaCl_2_ on SOD, POD, and CAT activity on *Zoysia* *japonica* leaves under drought. (A) SOD activity; (B) POD activity; (C) CAT activity. Vertical bars represent standard deviations (n=3). The different letters indicate significant difference among different treatments on a given day (p < 0.05, LSD multiple test). The asterisks indicate significant difference between given day and 0 d at the same treatment (p < 0.05, LSD multiple test).

The CaCl_2_ pretreatments had significant effects on antioxidant enzymes under drought conditions (Figure 6). SOD activity was increased in the 5 and 10 mM CaCl_2_ pretreatment groups, and there was no significant change in the 20 mM CaCl_2_ pretreatment group (Figure 6A). As drought was prolonged, SOD activity in the stressed leaves of all treatments increased. During drought stress, the SOD activity of the 10 mM CaCl_2_ pretreatment group was higher than that of the control, and that of the 20 mM CaCl_2_ pretreatment was lower. No significant difference in the SOD activity of the stressed leaves of the 5 mM CaCl_2_ pretreatment group and the control was observed (except at day 9). The 20 mM CaCl_2_ pretreatment group alone showed decreased POD activity (Figure 6B). Under drought conditions, the 10 mM CaCl_2_ pretreatment group showed significantly increased POD activity in comparison to the control. The 10 mM CaCl_2_ pretreatment group showed significantly increased CAT activity under no stress and drought stress (Figure 6C). The 20 mM CaCl_2_ pretreatment group exhibited reduced CAT activity after 9 days of drought.

### Changes in MDA and proline content

**Figure 7:**
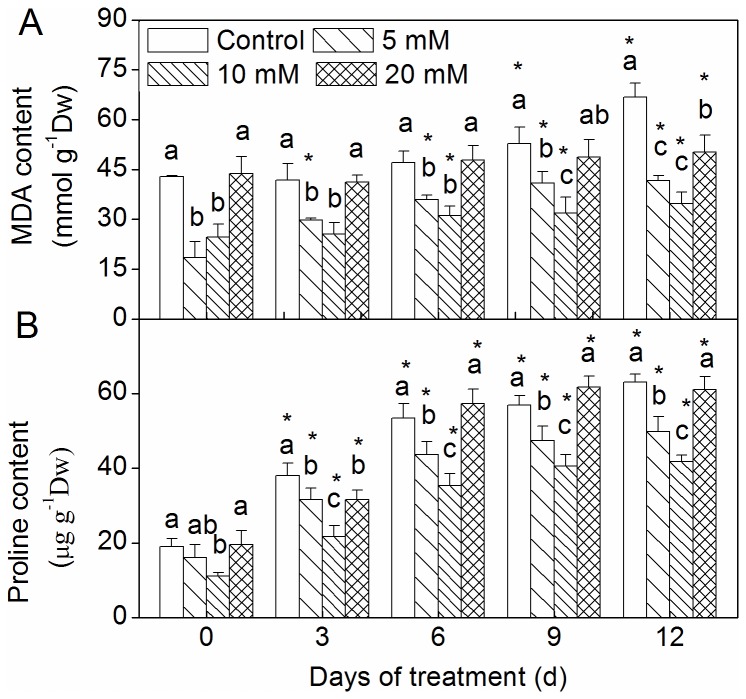
Effect of CaCl_2_ on MDA and proline contents on *Zoysia* *japonica* leaves under drought. (A) MDA content; (B) proline content. Vertical bars represent standard deviations (n=3). The different letters indicate significant difference among different treatments on a given day (p < 0.05, LSD multiple test). The asterisks indicate significant difference between given day and 0 d at the same treatment (p < 0.05, LSD multiple test).

MDA content showed an increasing trend under drought conditions (Figure 7A). Lower MDA content was recorded in the plants pretreated with 5 and 10 mM CaCl_2_ under no or drought stress. There was no significant change observed between the 20 mM CaCl_2_ pretreatment group and the control, with the exception of the change observed at 12 days after drought.

When subjected to progressive water stress, the proline content in zoysiagrass increased (Figure 7B), and the 5 and 10 mM CaCl_2_ pretreatment groups exhibited a significant decrease in proline accumulation in comparison to the control. The lowest proline content was observed in plants pretreated with 10 mM CaCl_2_.

## Discussion

The present investigation effectively confirmed that CaCl_2_ pretreatment could enhance the drought tolerance of zoysiagrass. Many studies have indicated that CaCl_2_ could act as a physiological treatment to increase plant tolerance. CaCl_2_ even at the relatively low concentrations used in this study, effectively contributed to the alleviation of drought stress.

Under stress conditions, the dry weight and Chl content are generally thought to be important parameters for growth measurement. In the present experiment, above- and below-ground fresh biomass and below-ground dry biomass increased in all CaCl_2_ pretreatment groups in comparison to the control (Figure 2). These results suggest that Ca^2+^ plays an important role in alleviating the damage to zoysiagrass incurred under drought conditions. Similar alleviation activity by applied CaCl_2_ has been reported for different plant species under different stress conditions [39–41]. Upadhyaya et al. [11] also reported that using foliar sprayed CaCl_2_ even after drought could increase the dry mass of leaves in the recovery phase. Increasing Ca^2+^ availability may reduce drought damage by increasing membrane integrity [19,42,43]. Our results showed a decrease in Chl content for all pretreatment groups and the control under drought (Figure 3). This decrease in Chl content under drought is due to the destruction of pigments and the instability of the pigment-protein complex [44]. The 5 and 10 mM CaCl_2_ pretreatment groups exhibited noticeably increased Chl content in the drought-stressed plants (Figure 3). It seems that the applied CaCl_2_ might prevent damage from cellular dehydration by balancing the osmotic strength of the cytoplasm [39]. In the present study, observed increases in biomass and Chl content values were greatest in the 10 mM CaCl_2_ pretreatment group (Figures 2, 3). The change in Pn for the 10 mM CaCl_2_ pretreatment group exhibited a similar trend (Figure 4). Further, similar results were reported by Amor et al. [24], who stated that the addition of 3.5 mM calcium was more effective than the addition of 20 mM in reducing NaCl stress for *Cakile* *maritima* .

Fv/Fm is an indicator of the efficiency of the photosynthetic apparatus [45]. In the present study, the Fv/Fm ratio was higher in plants pretreated with 10 mM CaCl_2_ than in other treatments (Figure 5). This demonstrates that the optimum CaCl_2_ concentration significantly enhances grass tolerance of drought without significantly affecting plant growth and development.

Oxidative stress is a key component of environmental stress, and increased SOD activity was correlated with increased protection from the damage associated with oxidative stress [7]. In the present study, SOD activity increased as drought was prolonged, and the SOD activity of the 10 mM CaCl_2_ pretreatment was higher than that of the control. The product of SOD activity is H_2_O_2_, which is still toxic and must be eliminated by conversion to H_2_O in subsequent reactions [24]. Some authors have indicated that the CAT/POD system might act cooperatively to remove H_2_O_2_ at a maximal rate and at a minimal expense of power reduction [27,46]. In fact, our results showed that POD activity of the 5 and 10 mM CaCl_2_ pretreatment groups increased compared to the control under drought conditions, and CAT activity of the 10 mM CaCl_2_ pretreatment group also increased. Interestingly, SOD, POD, and CAT activity levels of the 10 mM CaCl_2_ pretreatment were significantly higher than those of the control (Figure 6). This implies that an increase of antioxidant enzymes effectively scavenges ROS to provide protection from cellular oxidative damage. It has also been reported that external Ca^2+^ can induce significant increases in SOD, POD, and CAT activity in maize and cool season grasses seedlings [23,47].

We also observed that MDA contents in the leaves of pretreated and control groups gradually increased as drought continued (Figure 7A). According to Guimarães et al. [43], high MDA content may result in electrolyte leakage, indicating a loss of membrane integrity. However, the 5 and 10 mM CaCl_2_ pretreated sample groups showed lower MDA contents than did the control under drought. Some studies have shown that CaCl_2_ pretreatment decreased MDA content [20,47]. This result was in accordance with the increase in growth and antioxidant enzymes activities of zoysiagrass. This proved that pretreatment with the appropriate CaCl_2_ concentration alleviated and postponed oxidation damage that resulted from drought.

Free proline accumulation in the leaves under stress conditions is of utmost importance for plant adaptation during stress [48,49]. Our data suggests that proline content increased in zoysiagrass for all pretreatments under drought (Figure 7B). It was found that the availability of foliar CaCl_2_ could modulate endogenous proline accumulation under water stress, because the leaves of stressed plants that were pretreated with CaCl_2_ accumulated less proline than did the nontreated plants. Our results are consistent with those of Jaleel et al. (2007), who reported that addition of CaCl_2_ to drought stressed plants lowered the proline concentration by increasing the level of proline degrading enzyme and decreasing the proline synthesizing enzyme.

In summary, foliar application of CaCl_2_ can help zoysiagrass avoid the stress effects resulting from drought to some extent, as indicated by Chl content, Pn, chlorophyll fluorescence, antioxidant enzyme activities, lipid peroxidation, and proline content. These findings are very helpful for determining the ways in which conditions should be manipulated to secure the survival and improve the growth of turfgrass under drought conditions.
